# Organic Amendments Enhance Maize Growth by Improving Chemical and Microbial Properties in Coastal Saline–Alkali Soils

**DOI:** 10.3390/plants14142217

**Published:** 2025-07-17

**Authors:** Xiaoyu Huang, Tao Yin, Weijiao Sun, Guili Ge, Wenliang Wei

**Affiliations:** College of Resources and Environment, Qingdao Agricultural University, Qingdao 266109, China; xiaoyu122431@163.com (X.H.);

**Keywords:** maize, biochar, seaweed organic fertilizer, soil quality, microbial community, coastal saline–alkali soils

## Abstract

Biochar and seaweed fertilizers could improve soil quality and promote plant growth. However, the key soil factors and microbial mechanisms that drive maize growth in coastal saline–alkali soils remain unclear. A soil culture experiment was designed with four treatments—no organic fertilizer (CK), single seaweed fertilizer (F), single biochar (B), and combined application of seaweed fertilizer and biochar (BF)—to investigate the effects of biochar and seaweed fertilizer on maize growth and its mechanism. The results showed that B and BF significantly increased maize aboveground biomass by 8.86% and 17.28% compared to CK, respectively. The soil organic carbon, total nitrogen, available nitrogen, available phosphorus, available potassium content, and pH of B and BF were significantly increased. Bacterial diversity increased under B and BF, while fungal richness decreased under BF. The changes in the fungal community were mainly affected by soil available nitrogen, but there was no significant correlation between bacterial communities and these indicators. Pearson correlation analysis suggested that the bacterial Chao1 index was significantly positively correlated with maize growth indicators, soil available phosphorus, and available potassium, as well as the bacterial PD whole tree index with leaf area and available phosphorus. The fungal Shannon index was significantly negatively correlated with maize plant height, leaf area, SPAD, aboveground biomass, and soil total nitrogen and available nutrients. Overall, biochar and seaweed fertilization could significantly promote maize growth by improving soil chemical properties and microbial communities in coastal saline–alkali soils.

## 1. Introduction

In recent years, soil salinization has become more and more serious due to the excessive exploitation of groundwater and inappropriate irrigation and fertilization [1]. There are approximately 1.1 billion hectares of saline–alkali soil worldwide, with China accounting for 3.69 × 10^7^ hectares. It is widely distributed in coastal areas, inland arid regions, and the Huang–Huai–Hai Plain [2]. Saline–alkali soils are characterized by high pH and Na^+^, poor soil structure, depleted soil organic carbon (SOC), and low nutrient availability, which reduces crop productivity [3]. Increases in crop yield depend on healthy soil conditions [4]. Organic fertilizer application is one of the most important ways to improve the quality of saline–alkali soils [5]. Therefore, a study on a reasonable fertilization strategy to improve soil quality and crop yield is urgently needed.

Biochar is a kind of carbonaceous product made from organic waste via high-temperature pyrolysis [6,7]. Studies have illustrated that biochar application has a positive impact on the physical and chemical properties of saline–alkali soil. Biochar can adsorb soil ions through its inherent properties, including CEC, organic carbon content, and specific surface area, which collectively facilitate the leaching or retention of soil salt ions [8]. Biochar’s porous nature enhances water retention and ameliorates the physical structure of saline–alkali soils [9,10]. Wang et al. [11] demonstrated that biochar incorporation significantly improved the soil microenvironment, expanded the soil carbon pool, and had a stronger effect on carbon sequestration and emission reduction. Meanwhile, biochar application can delay fertilizer release, increase soil fertility sustainability, and increase plant growth and nutrient uptake in saline–alkali soils [12]. However, the results of biochar application are also controversial. Some studies have reported that too much biochar may reduce the abundance of the soil microbial community [13].

Seaweed fertilizers are characterized as safe, non-toxic, and environmentally friendly. Seaweed fertilizer application can promote the absorption of nutrients and the growth of plant cells and root development, improve the soil environment and microbial community and plant stress resistance, and enhance crop yield and quality [14]. Studies have shown that seaweed extract could alleviate drought stress by regulating the transpiration rate of crop plants and slow down the effect of salt stress on crops by enhancing the activities of catalase, superoxide dismutase, and ascorbate peroxidase [15]. Due to the fact that seaweed fertilizer is rich in protein, amino acids, and polysaccharides, its application could increase the abundance of many bacterial genera exhibiting functions related to polysaccharide degradation and nitrogen fixation, such as *Methylobacteria*, *Acidobacteria*, and *Micromonospora* [16]. The application of low concentrations of seaweed extracts had positive effects on seed germination, shoot growth, root growth, nutrient utilization efficiency, soil microorganisms, biotic stress, and abiotic stress [17].

At present, most of the studies on the effects of biochar application are distributed in tropical or subtropical regions [18], and the soil is mostly acid soil or nutrient-deficient sandy soil or sandy loam [19]; in this context, the effect of biochar application can usually be explained by soil pH, CEC, or soil water retention. Seaweed fertilizer is mainly studied based on its action on crops, including alleviating plant high-temperature stress, drought stress, the agronomic and biological effects of salt stress, and heavy metal stress [20]. There is research that shows that the combined application of biochar and seaweed fertilizer could improve the soil environment and crop productivity [21]. However, the key soil factors and microbial mechanisms that drive the growth of maize through the use of biochar and seaweed fertilizer in coastal saline–alkali soils remain unclear. Therefore, a soil culture experiment was conducted to explore the effects of biochar and seaweed fertilizer application on the growth of maize, soil chemical quality, and microbial community structure, as well as to analyze the key soil factors affecting maize growth. In this research, we aimed to (1) evaluate the effects of biochar and seaweed fertilizer application on maize growth promotion, soil quality enhancement, and microbial community structure modification and (2) identify the key factors responsible for enhanced maize productivity. The results will provide a theoretical basis and technical support for the green sustainable development of agriculture in coastal saline–alkali soils.

## 2. Results

### 2.1. Effects of Different Fertilization Treatments on Maize Growth Indicators and Nutrient Uptake

Fertilization treatments significantly improved maize growth indicators (Table 1). BF treatment showed the greatest enhancement, resulting in the highest PH, SD, LA, SPAD value, and AB. Compared to CK, BF improved PH, SD, LA, SPAD value, and AB by 67.00%, 19.23%, 33.70%, 18.82%, and 17.28%, respectively.

**Figure 1:**
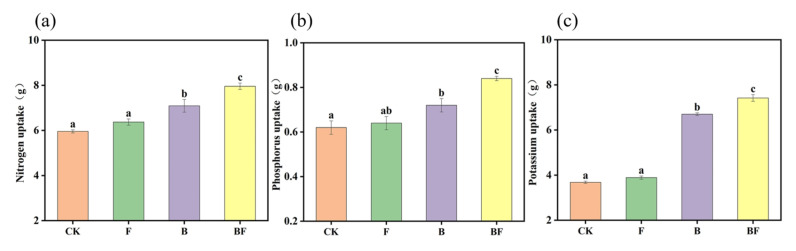
Effects of different fertilization treatments on maize nitrogen uptake (**a**), phosphorus uptake (**b**), and potassium uptake (**c**). CK: no organic fertilizer; F: single seaweed fertilizer; B: single biochar; and BF: combined application of seaweed fertilizer and biochar. Different lowercase letters indicate significant differences among fertilization treatments at *p* < 0.05.

Fertilization treatments enhanced maize nutrient uptake. BF significantly increased nitrogen (N) absorption by 33.56%, 24.96%, and 12.27% compared to CK, F, and B, respectively (Figure 1a). The values were 35.48% (CK), 31.25% (F), and 16.67% (B) for phosphorus (P) uptake (Figure 1b) and 101.63% (CK), 90.75% (F), and 10.75% (B) for potassium (K) uptake (Figure 1c).

### 2.2. Effects of Different Fertilization Treatments on Soil Chemical Properties

**Figure 2:**
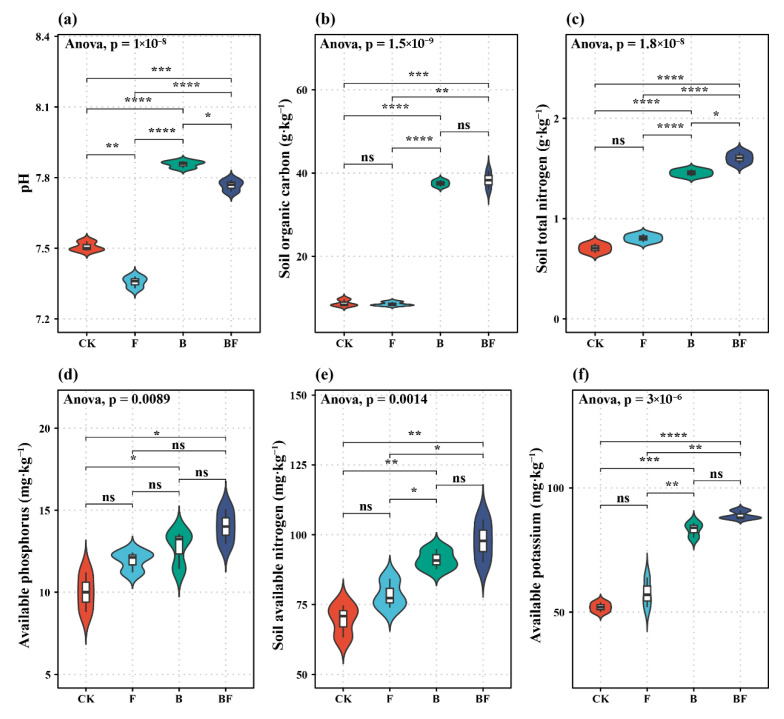
Effects of different fertilization treatments on soil pH (**a**), soil organic carbon (**b**), total nitrogen (**c**), available nitrogen (**d**), available phosphorus (**e**), and available potassium (**f**) content. CK: no organic fertilizer; F: single seaweed fertilizer; B: single biochar; and BF: combined application of seaweed fertilizer and biochar. A one-way ANOVA determined significant differences among the different treatments. A *t*-test was used to test differences among mean values of soil properties of different treatments. *, **, ***, and **** indicate statistically significant differences at *p* < 0.05, *p* < 0.01, *p* < 0.001, and *p* < 0.0001, respectively; ns indicates no significant difference.

Significant variations in soil properties across different fertilization treatments are shown in Figure 2. The pH values varied significantly among treatments, with B yielding the highest value, which was significantly higher than CK, F, and BF values of 4.66%, 6.79%, and 1.16%, respectively (Figure 1a). SOC also showed significant differences, with BF resulting in the highest content, which was significantly greater than CK, F, and B values of 337.33%, 346.50%, and 2.05%, respectively (Figure 2b). Similarly, TN was significantly different among treatments, with BF exhibiting the highest content, surpassing 128.57% (CK), 97.53% (F), and 9.59% (B) (Figure 2c). For AP, BF had the highest content, demonstrating values over 40.1% (CK), 17.53% (F), and 9.97% (B), which was significantly different from CK, while F and BF showed no significant differences (Figure 2d). AN followed the same trend, with BF showing the highest content, which was significantly different from CK, F, and B (Figure 2e). Lastly, AK was significantly higher in BF compared to CK, F, and B (Figure 2f).

### 2.3. Effects of Different Fertilization Treatments on Soil Microbial Community Composition

**Figure 3:**
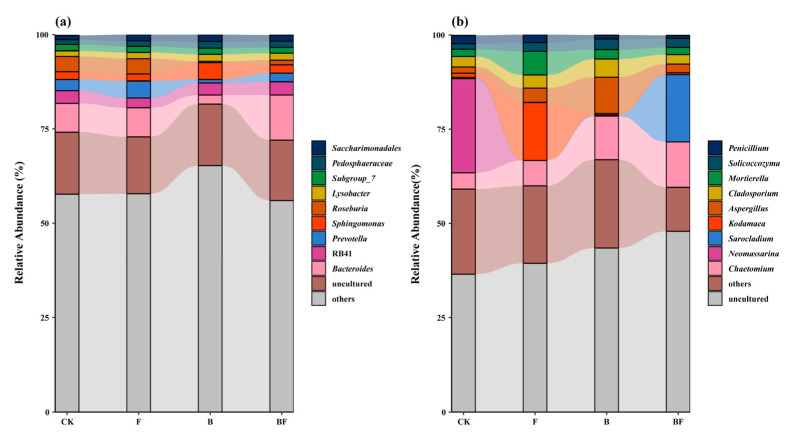
Effects of different fertilization treatments on the community composition of soil bacterial (**a**) and fungal (**b**) diversity at the genus level. CK: no organic fertilizer; F: single seaweed fertilizer; B: single biochar; and BF: combined application of seaweed fertilizer and biochar. Taxa not included in the top 10 are grouped into the “others” category.

Different fertilization treatments notably influenced soil bacterial and fungal composition (Figure 3). For the bacterial community, CK exhibited a high relative abundance of uncultured bacteria, comprising approximately 50% of the community. Seaweed fertilizer application resulted in a slight reduction in uncultured bacteria to around 45%, with an increased presence of *Saccharimonadales*, *Pedosphaeraceae*, and *Roseburia*. Biochar application further decreased uncultured bacteria to about 40%, enhancing the relative abundance of *Lysobacter* and *Sphingomonas*. BF yielded the most balanced bacterial community distribution, reducing uncultured bacteria to 35% and promoting diversity (Figure 3a).

For the fungal community, CK was dominated by unidentified fungi, accounting for 40%, along with *Penicillium* and *Solicoocczyma*. F decreased the unidentified fungi to 35%, while increasing *Cladosporium* and *Aspergillus*. B significantly diversified the fungal community, with *Mortierella*, *Kodamaea*, and *Chaetomium* showing increased relative abundances and unidentified fungi being reduced to 30%. BF further enhanced fungal diversity, prominently increasing *Aspergillus* and *Neomassari* and reducing unidentified fungi to 25% (Figure 3b).

### 2.4. Effects of Different Fertilization Treatments on Soil Microbial Richness and Diversity

**Figure 4:**
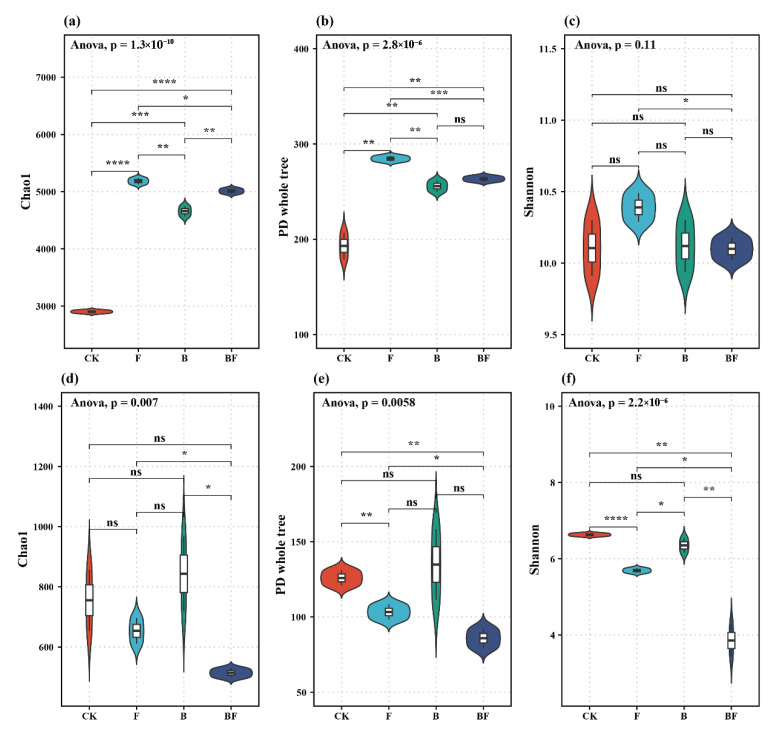
Effects of different fertilization treatments on soil bacterial (**a**–**c**) and fungal (**d**–**f**) richness and diversity index. CK: no organic fertilization; F: single seaweed fertilizer; B: single biochar; and BF: combined application of seaweed fertilizer and biochar. A *t*-test was used to test differences among the mean values of the microbial richness and diversity of different treatments. *, **, ***, and **** indicate statistically significant differences at *p* < 0.05, *p* < 0.01, *p* < 0.001, and *p* < 0.0001, respectively; ns indicates no significant difference.

Significant effects of different fertilization treatments on soil microbial richness and diversity were observed (Figure 4). For bacterial communities, the Chao1 index revealed a significant difference among treatments (*p* < 0.01), with BF showing the highest richness compared to CK (Figure 4a). Similarly, the PD whole tree index indicated significant differences, with BF exhibiting the greatest diversity (Figure 4b). Although the Shannon diversity index for bacterial communities did not show a significant overall difference, pairwise comparisons indicated higher diversity in F and BF compared to CK (Figure 4c). For fungal communities, the Chao1 index showed significant differences among treatments, with BF again achieving the highest richness (Figure 4d). PD whole tree index also showed significant differences, with BF having the highest index (Figure 4e). Finally, the Shannon diversity index revealed significant differences, with BF showing the highest diversity (Figure 4f).

### 2.5. Relationships Between Soil Microbial Communities, Soil Properties, and Maize Growth Indicators

**Figure 5:**
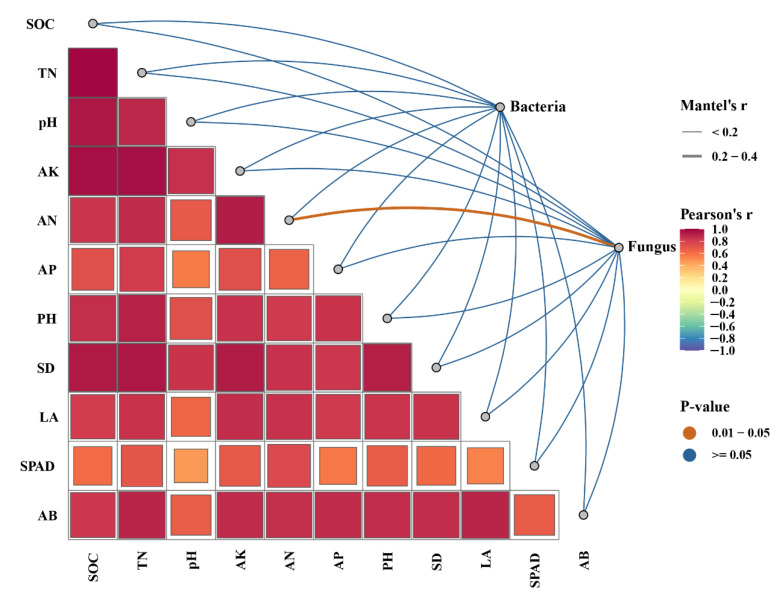
Relationships between soil bacterial and fungal community composition, maize growth indicators, and soil properties. AB, aboveground biomass; LA, leaf area; PH, plant height; SD, stem diameter; AN, available nitrogen; AP, available phosphorus; AK, available potassium; SOC, soil organic carbon; and TN, total nitrogen. Strong positive correlations are indicated by red, while negative correlations are shown in blue. Significant correlations (*p*-values between 0.01 and 0.05) are marked with orange dots, while non-significant correlations (*p*-values ≥ 0.05) are marked with blue dots. The network plot on the right side of the figure shows the correlations between environmental factors and the composition of bacterial and fungal communities, with line thickness representing Mantel’s r values (thin lines for r < 0.2 and thicker lines for r between 0.2 and 0.4).

Relationships between soil microbial community composition and maize growth indicators, as well as soil properties assessed through Mantel tests, are shown in Figure 5. The fungal community composition was significantly correlated with AN content. But there is no significant correlation between bacterial communities and these indicators.

**Figure 6:**
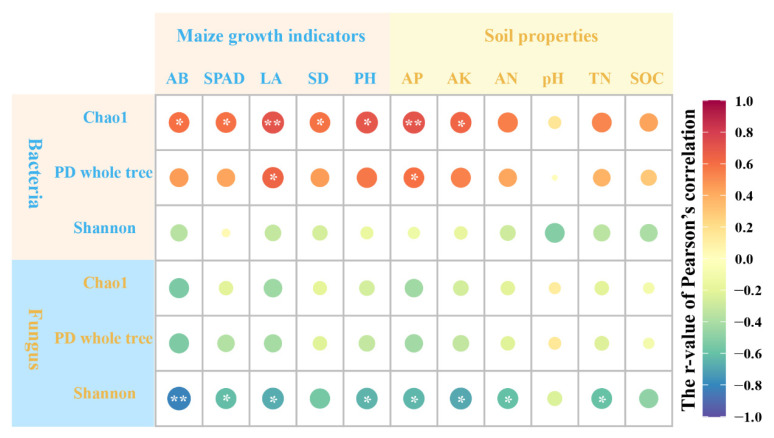
Pearson’s correlation analysis between soil microbial richness and diversity indexes, maize growth indicators, and soil properties. AB, aboveground biomass; LA, leaf area; PH, plant height; SD, stem diameter; AN, available nitrogen; AP, available phosphorus; AK, available potassium; SOC, soil organic carbon; and TN, total nitrogen. The size of the circle represents the size of the absolute value of the r value. * and ** indicate statistically significant differences at *p* < 0.05 and *p* < 0.01, respectively.

Pearson’s correlation analysis revealed significant relationships between microbial abundance and diversity, maize growth indicators, and soil properties (Figure 6). For bacterial communities, the Chao1 index showed significant positive correlations with AB, SPAD, SD, AK, and AN (*p* < 0.05), with particularly strong correlations with AP and AN (*p* < 0.001). Similarly, the PD whole tree index displayed significant correlations with AP (*p* < 0.05). However, the Shannon index did not show significant correlations with any measured variables. Additionally, the Shannon index for fungi displayed significant correlations with SPAD, LA, pH, AP, AK, AN, and TN, with the strongest correlations observed with AB (*p* < 0.01).

## 3. Discussion

### 3.1. Effects of Different Fertilization Treatments on Maize Growth

The results of this study demonstrated that the combined application of biochar and seaweed fertilizer exhibited the most pronounced effects on maize growth indicators and nutrient uptake, including PH, SD, LA, and SPAD values, as well as N, P, and K absorption (Table 1, Figure 1). The improvement in maize growth indicators could be primarily attributed to the improvements in both soil quality and microbial diversity [22]. These findings are consistent with the results reported by Ndiate et al. [23]. In detail, the mechanism underlying these improvements is likely attributed to the direct nutrient supplementation [24], as well as soil bulk density reduction, soil water enhancement, and buffering capacity improvement. Meanwhile, biochar application can significantly enhance soil properties and alter the structure of the bacterial community, which may serve as a key mechanism promoting crop growth [25,26]. Seaweed fertilizers are abundant in bioactive compounds, including polyphenols, lipids, proteins, and various amino acids derived from algal proteolysis; among these components, plant endogenous hormones such as brown algal polyphenols, fucoidan, betaines, cytokinins, auxins, and abscisic acid play a significant role in stimulating crop growth by promoting seed germination, root growth, and photosynthetic efficiency [27].

### 3.2. Effects of Different Fertilization Treatments on Soil Chemical Properties and Soil Microorganisms

The combined application of biochar and seaweed fertilizer enhanced SOC, TN, AP, and AK compared to CK (Figure 2). These findings align with the results of numerous previous studies. Biochar application has been reported to increase SOC [28] due to the high organic carbon content (686.3 g kg^−1^) and high C/N ratio (55.54:1), as well as the stability of biochar [6]. The increase in TN may be attributed to the fact that biochar application could alter chemical properties, thereby affecting the structure and activity of soil microbial communities involved in nitrogen fixation, nitrification, and denitrification, ultimately impacting soil TN content and enhancing soil AN availability [29]. Biochar application could significantly enhance soil AP content by mitigating soil phosphorus loss because biochar exhibits a significant adsorption capacity for phosphorus, and the sequestration of phosphorus via organic matter within biochar could effectively reduce phosphorus leaching risks [30]. Meanwhile, biochar application led to an increase in soil AK content [31]. In addition, biochar application increased soil pH (Figure 2), a finding that is consistent with the results of Buss et al. [32]. This phenomenon could be primarily attributed to two synergistic mechanisms: (1) the inherent alkalinity of the biochar matrix and (2) secondary mechanisms involving electrostatic interactions between weakly acidic functional groups (e.g., carboxyl moieties) on biochar’s surface and hydrogen ions, resulting in neutralization reactions that facilitate the release of exchangeable base cations—a biochemical process fundamental to soil pH amelioration [33].

Enhancing microbial diversity and improving microbial activity represent crucial strategies for optimizing the soil environment and increasing crop yield [34]. In this study, organic amendments were found to change the richness and diversity of soil microbial communities (Figure 4). The positive effects could be explained by previous studies, which reported that biochar could supply nutrients for microorganisms to enhance soil microbial biomass, create favorable microhabitats, and introduce labile organic compounds to promote the population of beneficial bacteria. The surface oxygen-containing functional groups present on biochar matrices mediate interspecies electron transfer between microbial consortia, effectively stimulating microbial metabolic processes through enhanced redox-mediated interactions and promoting synergistic microbial community dynamics [35,36]. Seaweed organic fertilizer application has also been confirmed to enhance the community structure and abundance of both soil bacteria and fungi [37].

The combined application of biochar and seaweed fertilizer was found to reduce the proportion of unidentified microbial communities [38]. We found that the relative abundance of uncultured soil bacteria was significantly reduced to 15%, and the proportion of unidentified soil fungi decreased by 15% (Figure 3), thereby improving the overall structure of microbial communities [39]. One possible explanation for decreasing fungal abundance may be related to the indirect changes in soil environments caused by biochar addition.

Additionally, the results of this study demonstrated that the increase in soil AP and AK significantly enhanced microbial community diversity (Figure 6), which aligns with findings from Widdig et al. [40]. Possible mechanisms may encompass the following dimensions: fertilization improved nutrient bioavailability, which promotes symbiotic relationships and co-evolution among microbial species, and fertilization enhanced the ecological adaptability of microbial communities, which increases the resistance of microbial populations to environmental stress and enhances their reproductive fitness [41]. Overall, the combined application of biochar and seaweed fertilizer effectively enhanced soil nutrient availability, improved soil microbial communities, and consequently promoted maize growth in coastal saline–alkali soils. However, given the limitations of pot experiments, further field experiments are required to validate these findings and evaluate their practical applicability. Meanwhile, the effects of a blank control and mineral N, P, and K application on soil properties should be further studied.

## 4. Materials and Methods

### 4.1. Experimental Site

A pot experiment was carried out in the research greenhouse at Qingdao Agricultural University, China (120°23′56.8″ E, 36°19′25.7″ N), from Oct. to Nov. 2023 (in total, for about 45 days). Growth conditions included a 20°C temperature, 60% relative humidity, and adequate light. The soil was collected from Changyi, which belongs to coastal saline–alkali soil. The main soil chemical properties (0–20 cm) were soil organic carbon (SOC) 9.85 g kg^−1^, total nitrogen (TN) 0.70 g kg^−1^, available nitrogen (AN) 84.28 mg kg^−1^, available phosphorus (AP) 9.92 mg kg^−1^, available potassium (AK) 57.36 mg kg^−1^, pH (soil-to-water ratio = 1:2.5) 7.46, and soil soluble salt 2.57‰. Soil texture was lightly loamy (50.21% sand, 29.87% silt, and 19.92% clay).

### 4.2. Experimental Design

Four treatments were set up in the experiment: (1) no organic fertilizer (CK), (2) single seaweed fertilizer (F), (3) single biochar (B), and (4) the combined application of seaweed fertilizer and biochar (BF). Three replicates per treatment were carried out for a total of 12 potted plants. Maize was planted in resin pots (upper diameter 18.5 cm, lower diameter 13 cm, and height 16 cm) on the ground. The same amounts of nitrogen, phosphorus, and potassium fertilizer were applied to 2 kg of soil, including nitrogen fertilizer (urea, N, 46%) 0.77 g, phosphate fertilizer (superphosphate, P_2_O_5_, 16%) 0.40 g, and potassium fertilizer (potassium sulfate, K_2_O, 50%) 1.10 g for each pot. The amounts of biochar and seaweed fertilizers were 60.00 g and 2.31 g, respectively. Biochar feedstock consisted of 70% maize straw and 30% sawdust and was pyrolyzed at 450–500°C in a commercial-scale reactor, wherein conditions of organic carbon 683.1 g kg^−1^, nitrogen 12.3 g kg^−1^ (C/N = 55.54), phosphorus 1.3 g kg^−1^, potassium 4.0 g kg^−1^, and pH 8.3 for biochar were included. Seaweed fertilizer contained organic carbon 203.2 g kg^−1^, nitrogen 34.1 g kg^−1^ (C/N = 5.96), phosphorus 10.0 g kg^−1^, and potassium 57.8 g kg^−1^. The pot experiment was carried out after germination of the maize seeds. The soil and fertilizers were evenly mixed and placed into the basin. Each pot scattered three maize seeds (the maize variety was Zhengdan 958), and then a graduated cylinder was used to pour a sufficient amount of the same volume of water. After the maize emerged from the seed, only one plant per pot was kept. Then, the amounts of water to be added were determined based on the growth conditions and kept consistent. Maize was harvested at the six-leaf stage.

### 4.3. Sampling and Determination

Plant height (PH), stem diameter (SD), leaf area (LA), and SPAD value were measured at the maize six-leaf stage. PH was measured with a ruler, SD was measured with a vernier caliper, and the SPAD value of the newly fully expanded leaves was measured with a SPAD–502 Plus portable chlorophyll analyzer. The length coefficient method was used to determine the LA of the newly fully expanded leaf, and the coefficient is 0.75 [42]. Subsequently, plant samples were placed and dried until they achieved a constant weight at 75°C. Then, nutrients (N/P/K) were measured via H_2_SO_4_–H_2_O_2_ digestion.

Soil samples were collected by removing roots after the aboveground harvest. Soil indicators, including soil pH, SOC, TN, AN, AP, and AK, were measured. Among them, SOC was determined by the potassium dichromate volumetric method, TN was determined by the semi-micro-Kelvin method, AN was determined by the alkaline hydrolysis diffusion method, AP was determined by the sodium bicarbonate method, and AK was determined by the ammonium acetate flame photometric method. The pH value (soil-to-water ratio = 1:2.5) was determined by the potentiometric method [43].

Soil microbial genomic DNA was extracted from 0.5 g soil samples using a FastDNA Spin kit for soil (MOBIO PowerSoil DNA Isolation Kit) according to the manufacturer’s instructions. For the PCR amplification of the partial 16S rRNA gene, two primers were used as follows: 515F (5′–barcode–GTGCCAGCMGCCGCGG–3′) and 907R (5′–CCGTCAATTCMTTTRAGTTT–3′). The DNA amplicons were detected by electrophoresis in a 2% agarose gel and were then purified using an AxyPrep DNA Gel Extraction kit (Axygen Biosciences, Union City, CA, USA) according to the manufacturer’s instructions. They were subsequently quantified using a QuantiFluorTM–ST fluorescent quantitative system (Promega, Madison, WI, USA). Purified amplicons were directly used for paired-end sequencing (2 × 250) on an Illumina MiSeq platform (MajorBio Shanghai Technologies, Shanghai, China) according to standard protocols [44].

### 4.4. Statistical Analysis

Data were expressed as mean ± standard deviation. A one-way analysis of variance was used to determine the significant differences in soil chemical properties and maize growth indicators among different fertilization treatments, and the abundance and diversity of bacterial and fungal communities were weighted by PCoA. Microsoft Excel 2021, SAS (version 8.2; SAS Institute Inc., Cary, NC, USA), and PCoA were used for data collation and statistical analysis. The relationships between microbial community composition, maize growth indicators, and soil properties were examined in R (version 4.4.1; https://www.R-project.org/, accessed on 14 June 2024) using ggcor and linkET packages. The relationships between alpha diversity, maize growth indicators, and soil properties were analyzed in R using the psych package. R language tools and Sigma Plot (version 12.0; SysTest Software Inc., San Jose, CA, USA) software mapping were also used for analysis.

## 5. Conclusions

The results demonstrated that the combined application of biochar and seaweed fertilizer significantly promoted maize growth, enhanced soil nutrient contents, and increased the richness and diversity of microbial communities. Meanwhile, the improvement in soil chemical properties induced by fertilization also played an important role in increasing soil microbial community diversity and promoting maize growth. In conclusion, the combined application of biochar and seaweed fertilizer is an important fertilizer application measure to promote crop growth and improve soil nutrient supply and micro-ecological quality in coastal saline–alkali soils.

## Figures and Tables

**Table 1:** Effects of different fertilization treatments on maize growth indicators.

	PH (cm)	SD (cm)	LA (cm^2^)	SPAD	AB (g Plant^−1^)
CK	33.58 ± 1.45 c	1.30 ± 0.01 b	117.77 ± 8.56 c	34.28 ± 2.41 b	4.63 ± 0.07 c
F	42.25 ± 4.02 b	1.36 ± 0.03 b	134.88 ± 2.98 b	37.18 ± 1.47 ab	4.80 ± 0.04 c
B	52.00 ± 0.14 a	1.54 ± 0.02 a	147.83 ± 0.39 ab	38.85 ± 0.78 ab	5.04 ± 0.09 b
BF	56.08 ± 0.74 a	1.55 ± 0.01 a	157.46 ± 1.97 a	40.73 ± 0.68 a	5.43 ± 0.02 a

Data are expressed as mean ± standard deviation. CK, no organic fertilizer; F, single seaweed fertilizer; B, single biochar; and BF, combined application of seaweed fertilizer and biochar. PH, plant height; SD, stem diameter; LA, leaf area; and AB, aboveground biomass. Different lowercase letters indicate significant differences among fertilization treatments at *p* < 0.05.

## Data Availability

Data are contained within the article.
